# Supplementary material for: Coinfection frequency in water flea populations is a mere reflection of parasite diversity
Source: Commun Biol. 2024 May 11;7:559. doi: 10.1038/s42003-024-06176-8 (PMC11088698; doi:10.1038/s42003-024-06176-8)
Supplement: Supplementary file 2 — Supplementary Figures [file 42003_2024_6176_MOESM2_ESM.pdf]

Coinfection frequency in water flea populations is a mere reflection of parasite diversity.

Supplementary Figures

Snir Halle[1], Ofir Hirshberg[1], Florent Manzi[2], Justyna Wolinska[2,3] and Frida Ben-Ami[1].

[1] School of Zoology, George S. Wise Faculty of Life Sciences, Tel-Aviv University, Tel-Aviv 6997801, Israel.

[2] Department of Evolutionary and Integrative Ecology, Leibniz Institute of Freshwater Ecology and Inland Fisheries, Berlin, Germany.

[3] Department of Biology, Chemistry, Pharmacy, Institute of Biology, Freie Universität Berlin, Berlin, Germany.

Corresponding author: Snir Halle, email: snirhalle@mail.tau.ac.il.

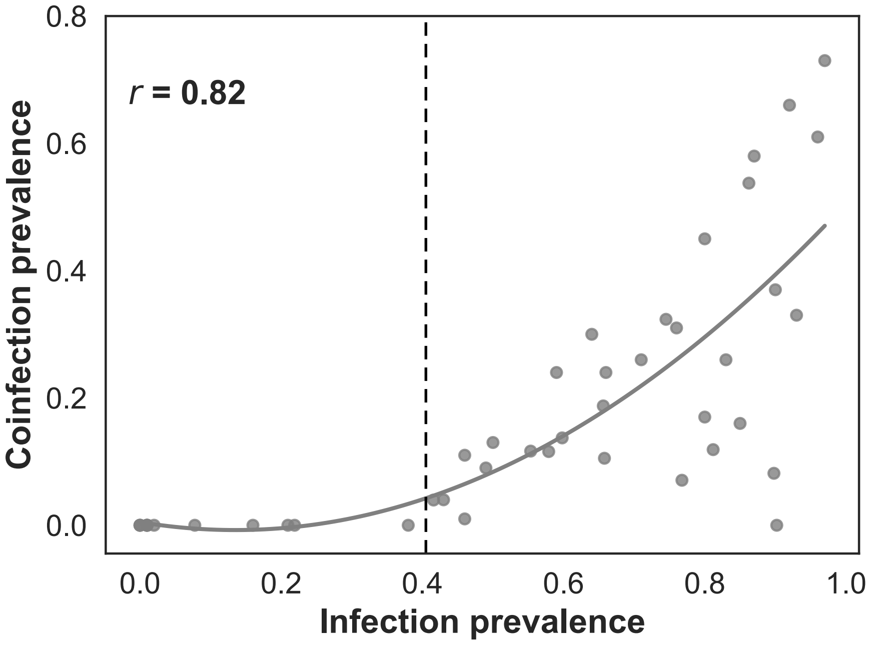

Figure S1. The prevalence of coinfections plotted against the general prevalence of infection in the population. A dashed line represents the infection prevalence below which no coinfections were observed. The Spearman correlation value highlights the tight but non-linear relation between these variables.

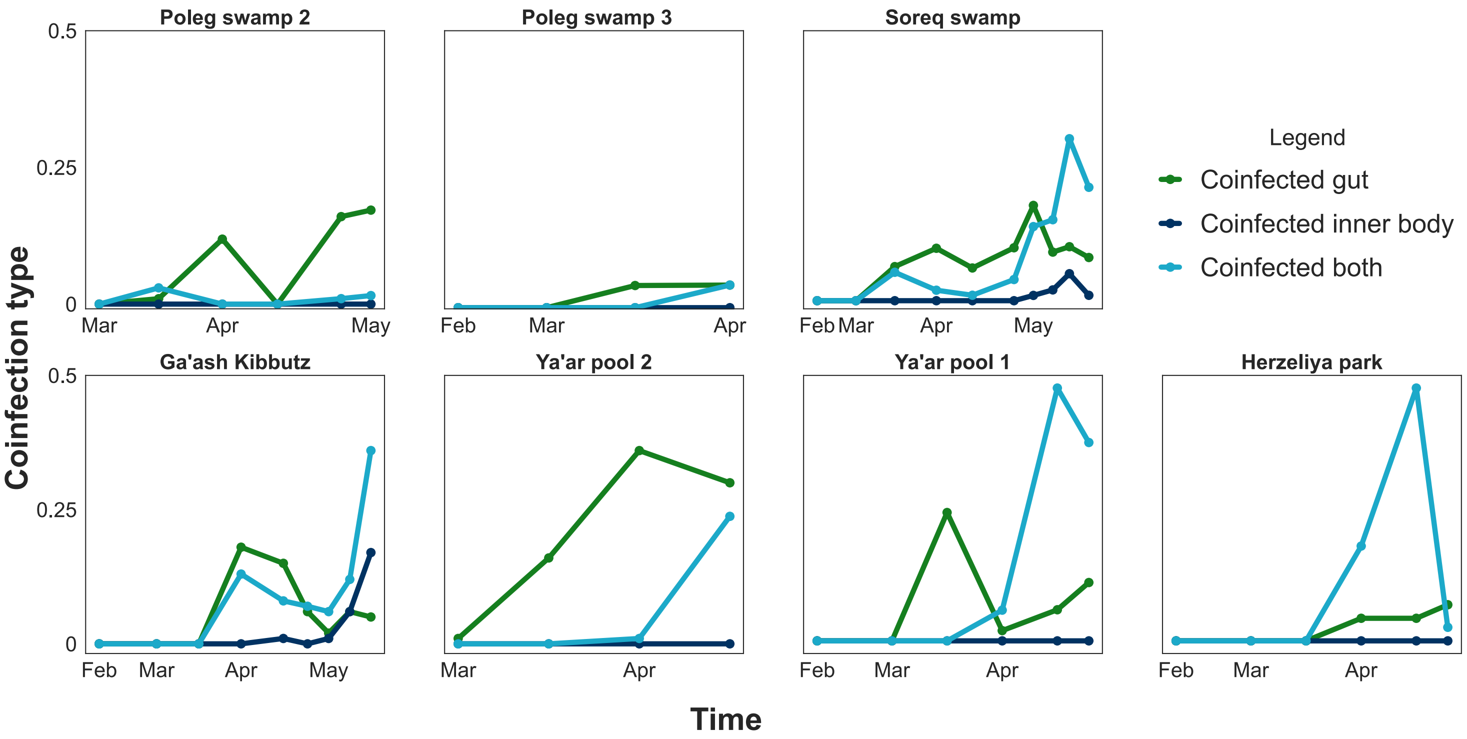

Figure S2. Proportions of coinfection types in the sampled populations over time. Coinfection that occurred solely in the gut is represented by a green line, solely in the inner body by a blue line and in both sites by a cyan line. Dots represent the sampling dates.

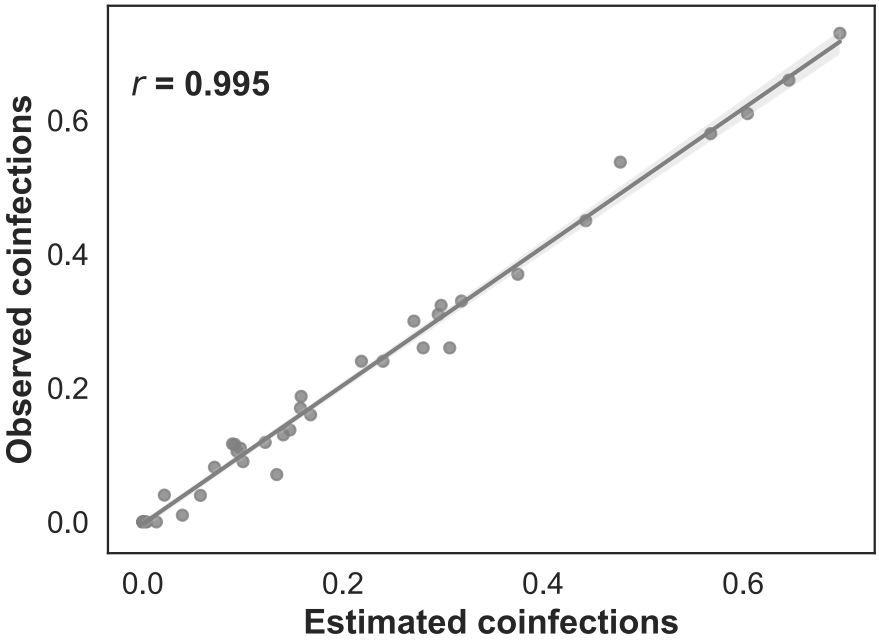

Figure S3. The correlation between the observed prevalence of coinfection in every sample and their respective estimated prevalence by the null model.

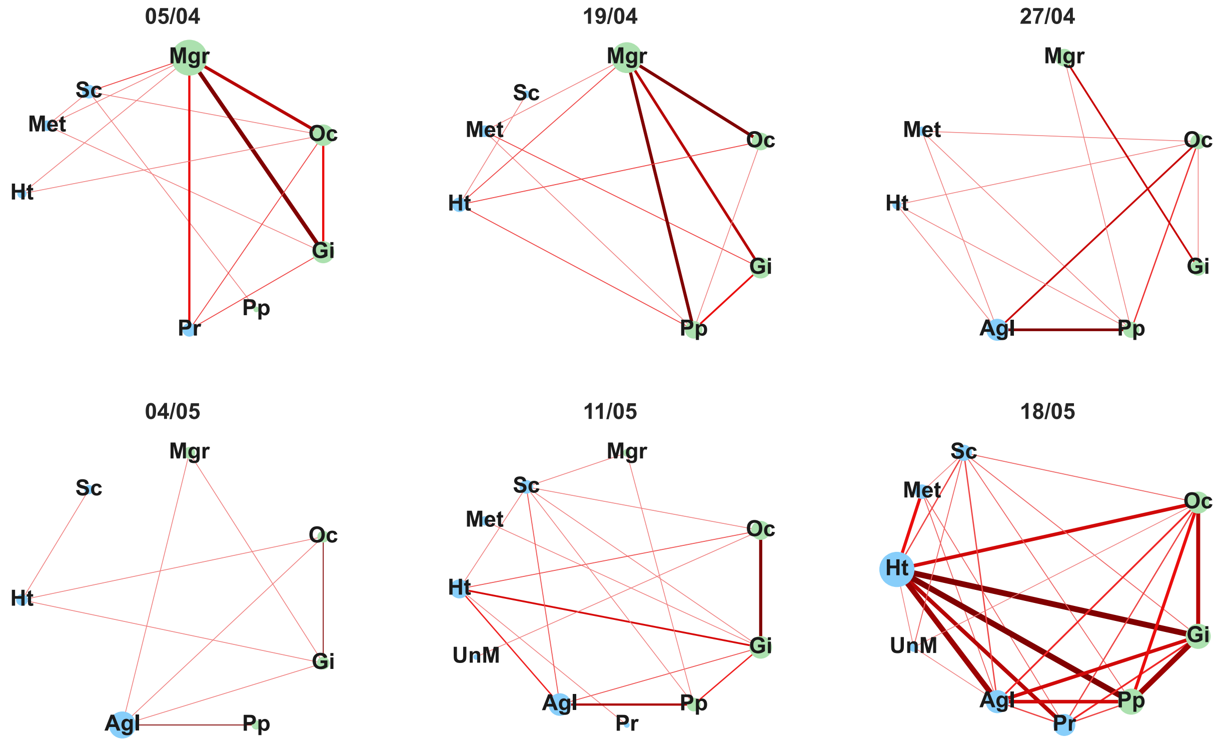

Figure S4. Parasite infection networks in Ga'ash Kibbutz throughout the entire season. The date of the respective sample is presented on the top of each network. Nodes, i.e., parasite species, are represented by circles with species abbreviations. The prevalence of the different species is represented by the relative size of the circle. Edges, i.e., coinfecting pairs, are ranked relatively by color gradient and thickness of the line (edge weight), with lines becoming darker and thicker as a pair becomes more frequent. Parasitic groups are designated by different node color, green or blue for gut or inner body parasites, respectively. Species abbreviations are as follows: Agl- *Aglomerata* sp., Gi- *Glugoides intestinalis*, Ht- *Hamiltosporidium tvaerminnensis*, Met- *Metschnikowia bicuspidata*, Mgr- Unfamiliar species of microsporidium infecting the host guts, Oc- *Ordospora colligata*, Pp- *Pansporella perplexa*, Pr-*Pasteuria ramosa*, Sc- *Spirobacillus cienkowskii*, UnM- represents the pooled category of unknown microsporidia species.

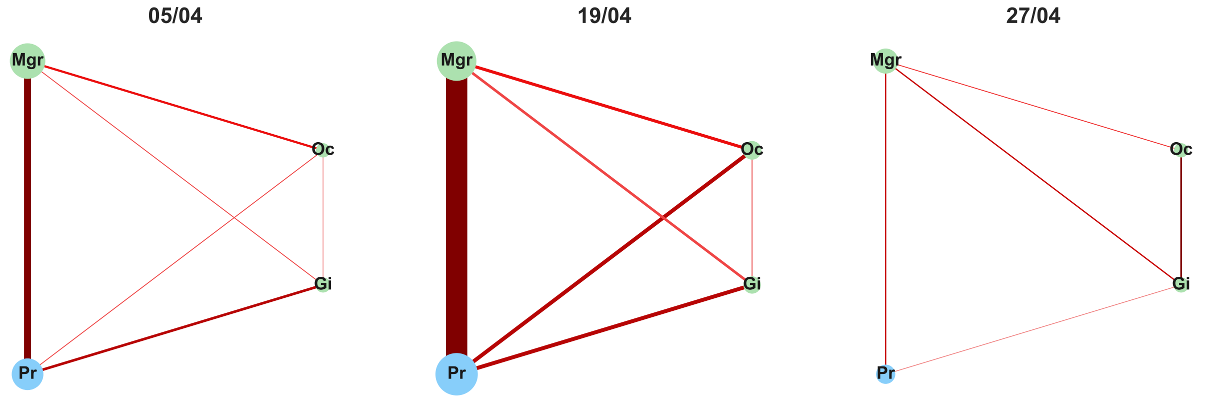

Figure S5. Parasite infection networks in Herzliya park throughout the entire season. The date of the respective sample is presented on the top of each network. Nodes, i.e., parasite species, are represented by circles with species abbreviations. The prevalence of the different species is represented by the relative size of the circle. Edges, i.e., coinfecting pairs, are ranked relatively by color gradient and thickness of the line (edge weight), with lines becoming darker and thicker as a pair becomes more frequent. Parasitic groups are designated by different node color, green or blue for gut or inner body parasites, respectively. Species abbreviations are as follows: Gi- *Glugoides intestinalis*, Mgr- Unfamiliar species of microsporidium infecting the host guts, Pr- *Pasteuria ramosa*.

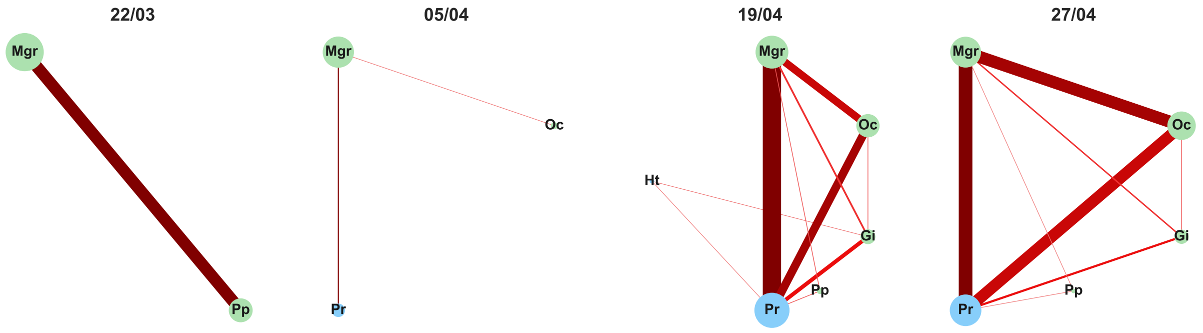

Figure S6. Parasite infection networks in Ya'ar pool 1 throughout the entire season. The date of the respective sample is presented on the top of each network. Nodes, i.e., parasite species, are represented by circles with species abbreviations. The prevalence of the different species is represented by the relative size of the circle. Edges, i.e., coinfecting pairs, are ranked relatively by color gradient and thickness of the line (edge weight), with lines becoming darker and thicker as a pair becomes more frequent. Parasitic groups are designated by different node color, green or blue for gut or inner body parasites, respectively. Species abbreviations are as follows: Gi- *Glugoides intestinalis*, Ht- *Hamiltosporidium tvaerminnensis*, Mgr- Unfamiliar species of microsporidium infecting the host guts, Oc- *Ordospora colligata*, Pp- *Pansporella perplexa*, Pr- *Pasteuria ramosa*.

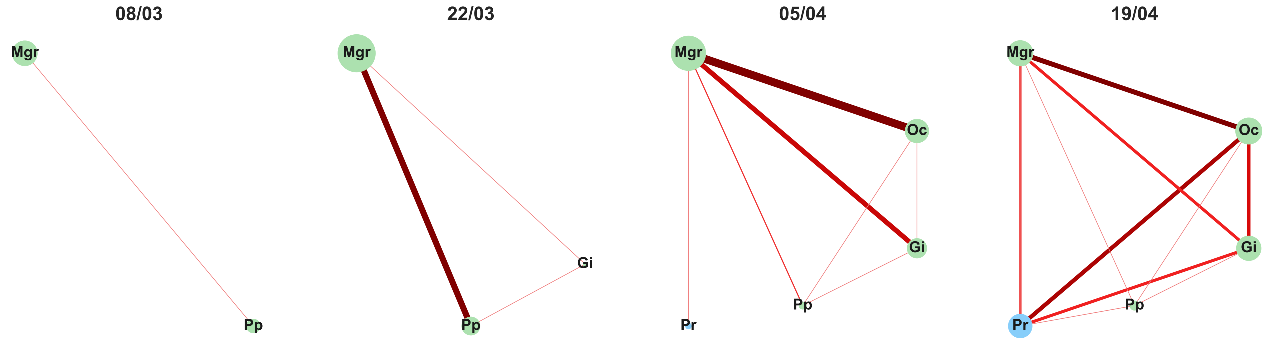

Figure S7. Parasite infection networks in Ya'ar pool 2 throughout the entire season. The date of the respective sample is presented on the top of each network. Nodes, i.e., parasite species, are represented by circles with species abbreviations. The prevalence of the different species is represented by the relative size of the circle. Edges, i.e., coinfecting pairs, are ranked relatively by color gradient and thickness of the line (edge weight), with lines becoming darker and thicker as a pair becomes more frequent. Parasitic groups are designated by different node color, green or blue for gut or inner body parasites, respectively. Species abbreviations are as follows: Gi- *Glugoides intestinalis*, Mgr- Unfamiliar species of microsporidia infecting the host guts, Oc- *Ordospora colligata*, Pp- *Pansporella perplexa*, Pr- *Pasteuria ramosa*.

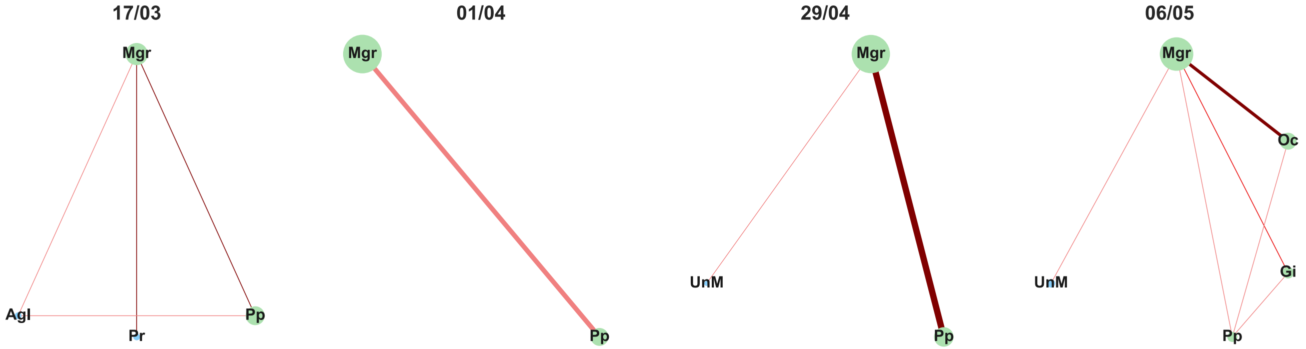

Figure S8. Parasite infection networks in Poleg swamp 2 throughout the entire season. The date of the respective sample is presented on the top of each network. Nodes, i.e., parasite species, are represented by circles with species abbreviations. The prevalence of the different species is represented by the relative size of the circle. Edges, i.e., coinfecting pairs, are ranked relatively by color gradient and thickness of the line (edge weight), with lines becoming darker and thicker as a pair becomes more frequent. Parasitic groups are designated by different node color, green or blue for gut or inner body parasites, respectively. Species abbreviations are as follows: Agl- *Aglomerata* sp., Gi- *Glugoides intestinalis*, Mgr- Unfamiliar species of microsporidium infecting the host guts, Oc- *Ordospora colligata*, Pp- *Pansporella perplexa*, Pr- *Pasteuria ramosa*, UnM- represents the pooled category of unknown microsporidia species.

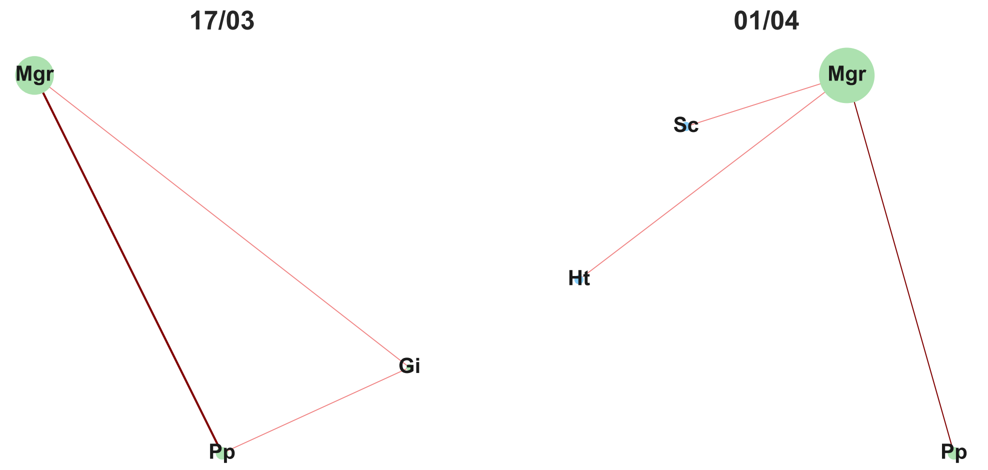

Figure S9. Parasite infection networks in Poleg swamp 3 throughout the entire season. The date of the respective sample is presented on the top of each network. Nodes, i.e., parasite species, are represented by circles with species abbreviations. The prevalence of the different species is represented by the relative size of the circle. Edges, i.e., coinfecting pairs, are ranked relatively by color gradient and thickness of the line (edge weight), with lines becoming darker and thicker as a pair becomes more frequent. Parasitic groups are designated by different node color, green or blue for gut or inner body parasites, respectively. Species abbreviations are as follows: Gi- *Glugoides intestinalis*, Ht- *Hamiltosporidium tvaerminnensis*, Mgr- Unfamiliar species of microsporidium infecting the host guts, Pp- *Pansporella perplexa*, Pr- *Pasteuria ramosa*, Sc- *Spirobacillus cienkowskii*.

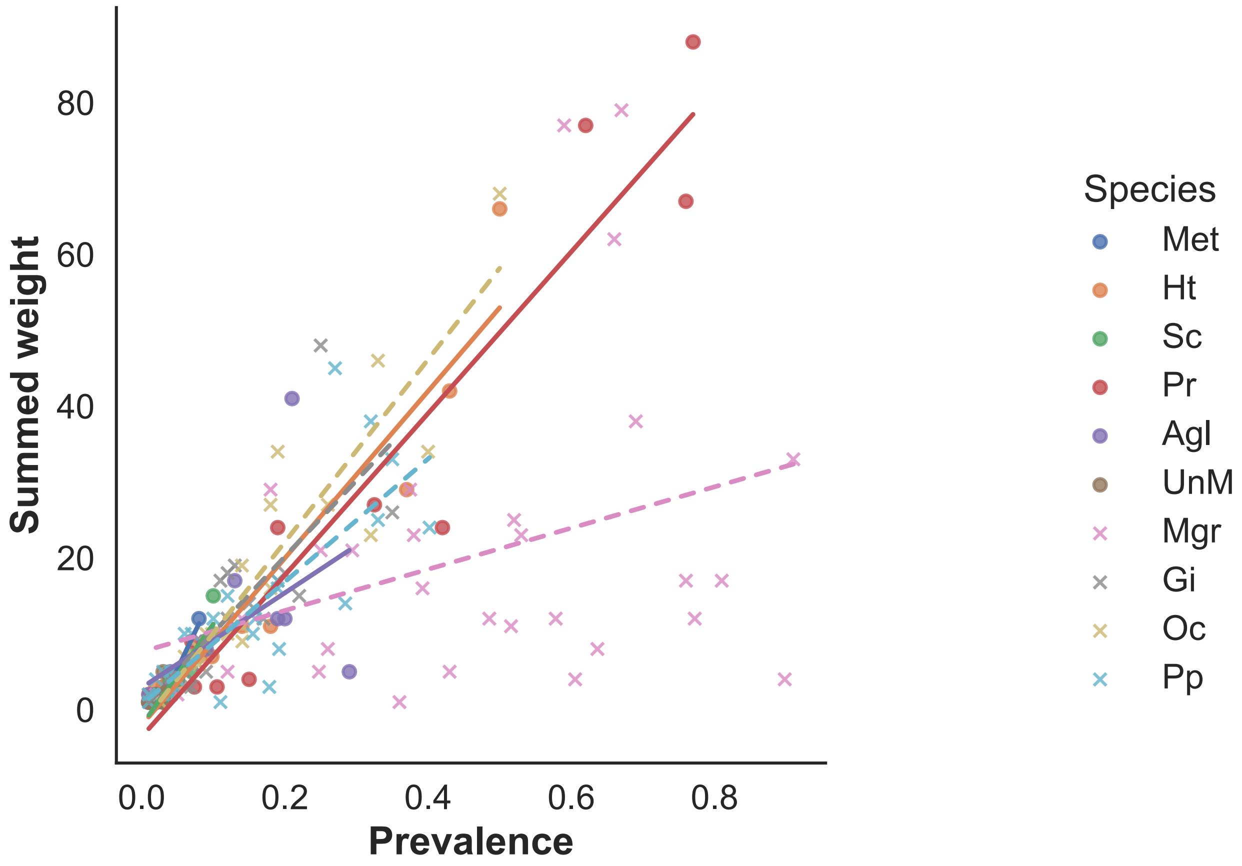

Figure S10. The correlation between species sums of weights (i.e., the number of times they were in coinfection) against their prevalence. Circles and diamonds differentiate between inner body and gut parasites, respectively. Species abbreviations are as follows: Agl- *Aglomerata* sp., Gi- *Glugoides intestinalis*, Ht- *Hamiltosporidium tvaerminnensis*, Met- *Metschnikowia bicuspidata*, Mgr- Unfamiliar species of microsporidium infecting the host guts, Oc- *Ordospora colligata*, Pp- *Pansporella perplexa*, Pr- *Pasteuria ramosa*, Sc- *Spirobacillus cienkowskii*, UnM- represents the pooled category of unknown microsporidia species.

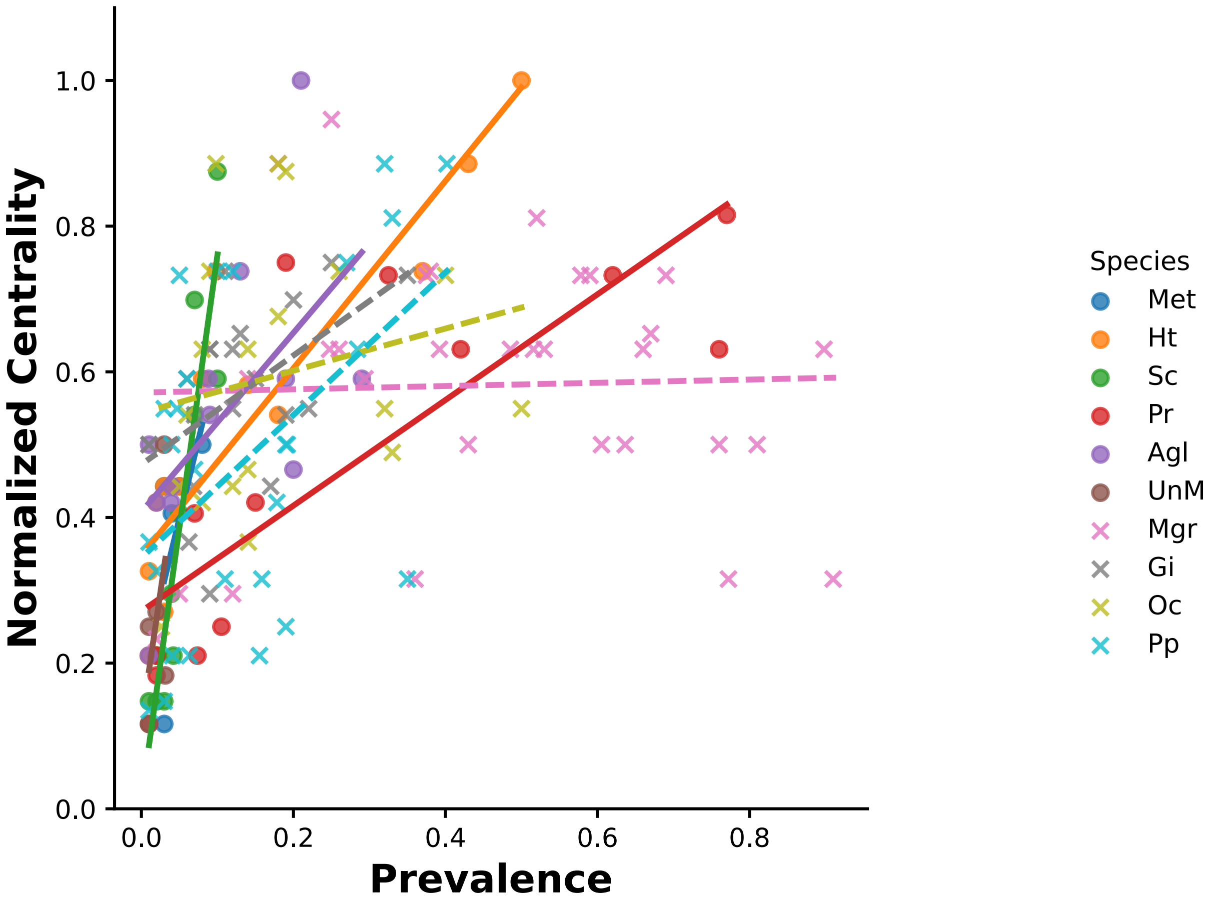

Figure S11. The correlation between species normalized centrality, i.e., the degree centrality of a node (proportion of connections within the network) penalized by the respective parasite diversity, against their prevalence. Circles and diamonds differentiate between inner body and gut parasites, respectively. Species abbreviations are as follows: Agl- *Aglomerata* sp., Gi- *Glugoides intestinalis*, Ht- *Hamiltosporidium tvaerminnensis*, Met- *Metschnikowia bicuspidata*, Mgr- Unfamiliar species of microsporidium infecting the host guts, Oc- *Ordospora colligata*, Pp- *Pansporella perplexa*, Pr- *Pasteuria ramosa*, Sc- *Spirobacillus cienkowskii*, UnM- represents the pooled category of unknown microsporidia species.

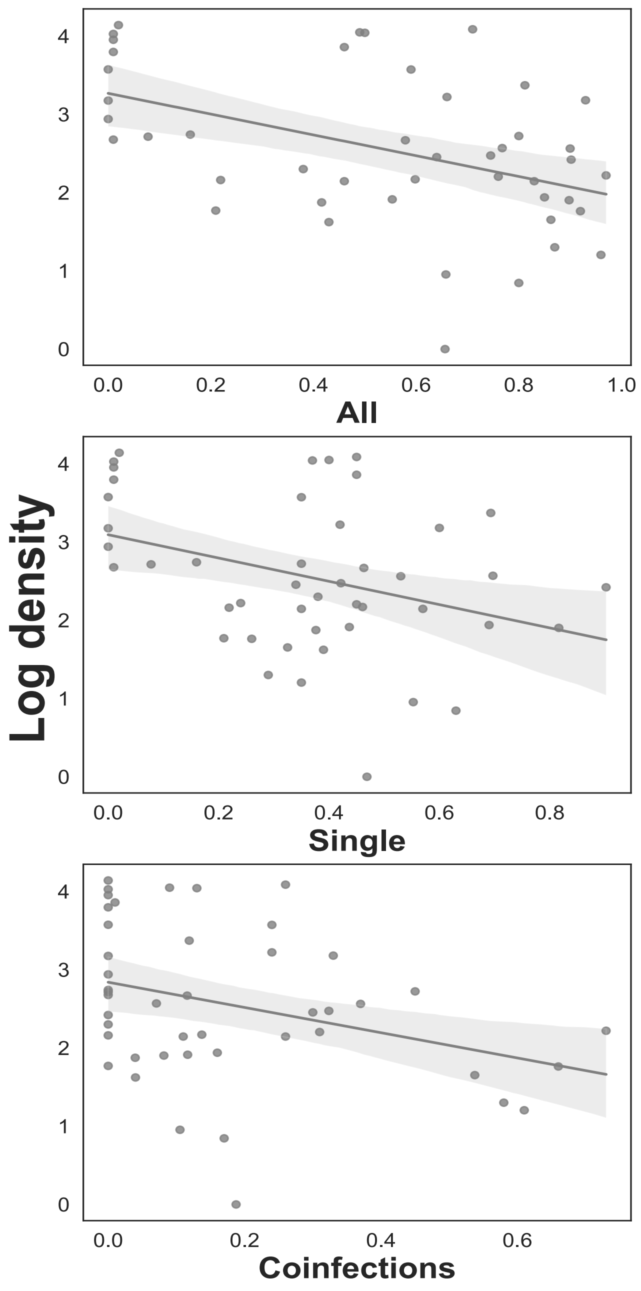

Figure S12. Regression plots of host density (log transformed) against the prevalence of all infection types (top panel), infections by a single parasite species (central panel) and coinfections (bottom panel).

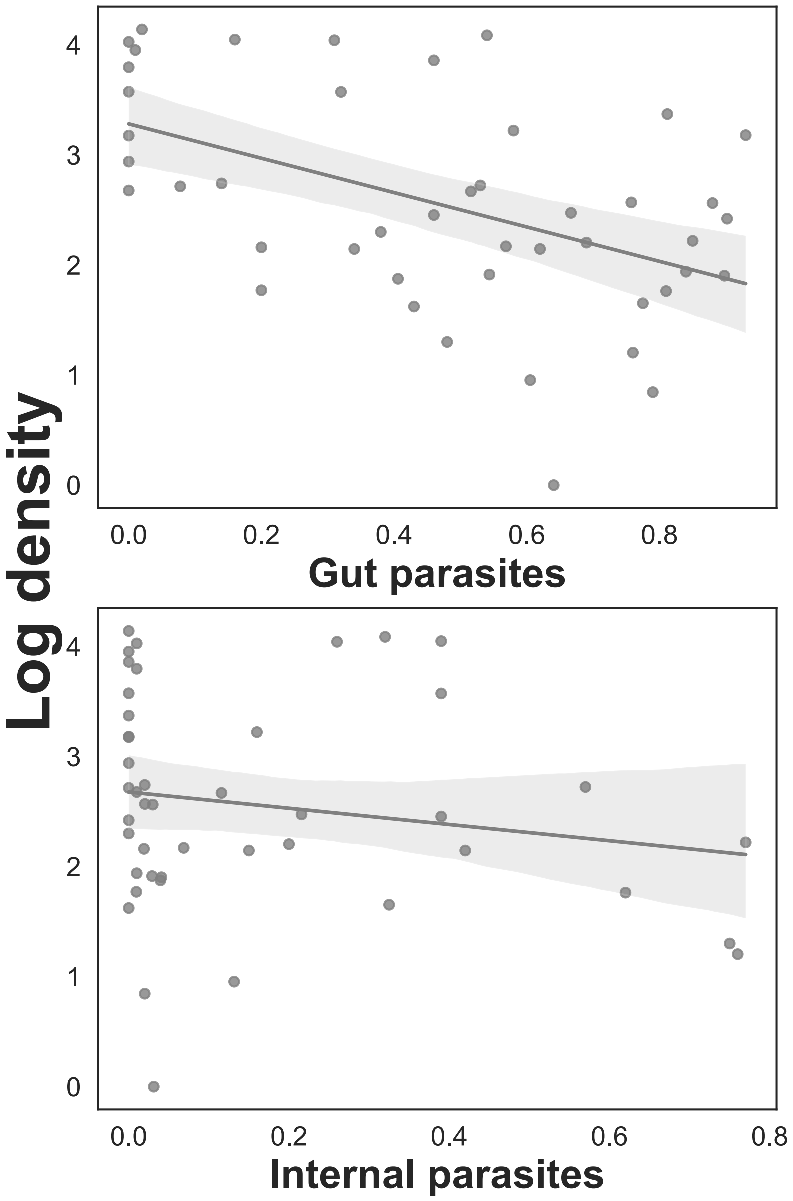

Figure S13. Regression plots of host density (log transformed) against the prevalence of gut parasites (top panel) and internal parasites (bottom panel).

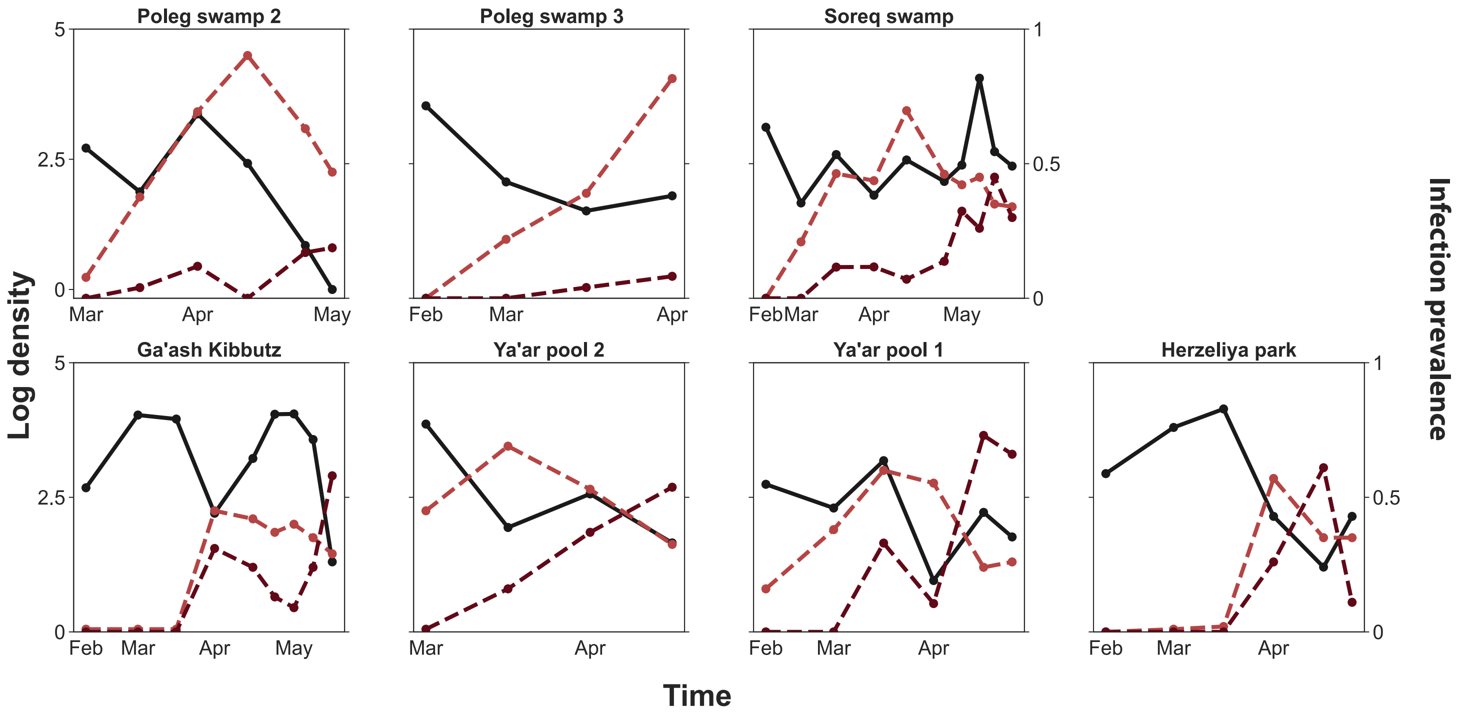

Figure S14. The development of host density (log transformed) throughout the season is presented (black lines) for each population along the prevalence of infection by single parasite species and coinfections (red and dark red dashed-lines, respectively).

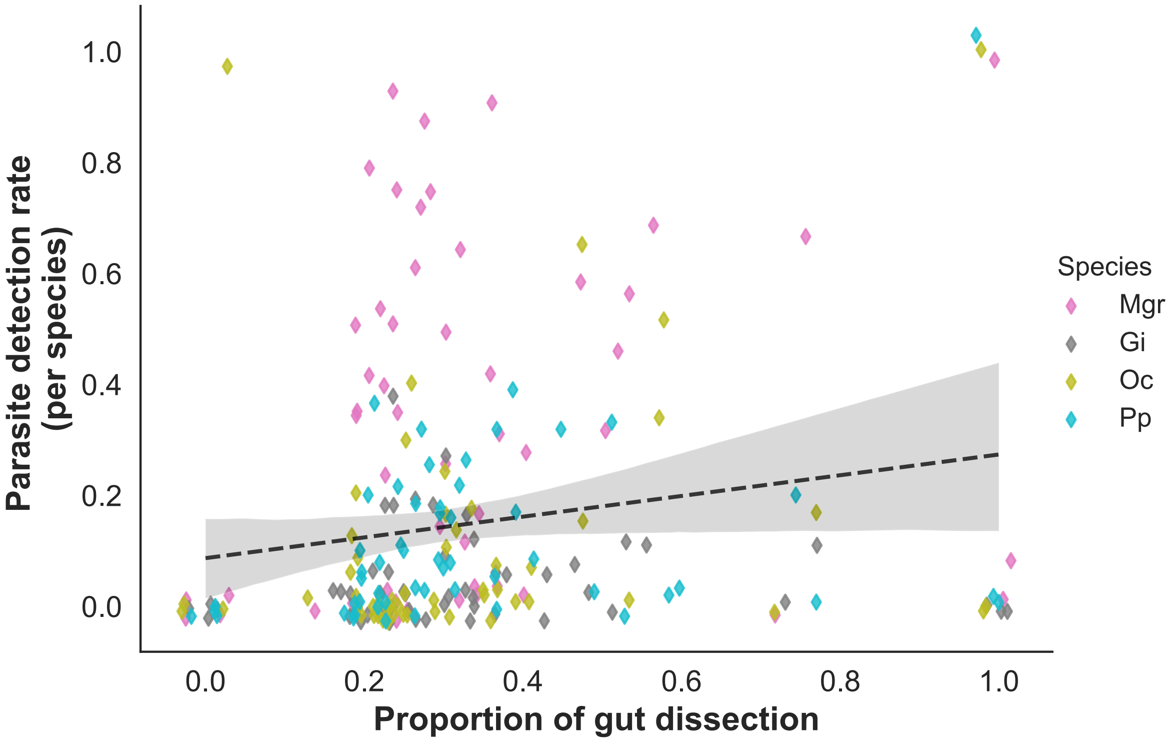

Figure S15. The relationship between the rate of gut dissection and the identification of gut parasites. Diamonds represent the scatter of the data for each species, while the regression line represents the general correlation of all the species together. The correlation between gut dissection and the ability to detect gut parasites was low (Pearson r= 0.17, p= 0.01, n= 216) and even lower when removing samples with extreme cases of 0% and 100% of gut dissection (Pearson r= 0.11, p= 0.11, n= 188). Therefore, we deduced that the rate of false negative for gut parasites detection was low and that their prevalence was reliably estimated.
