# Supplementary material for: Coinfection frequency in water flea populations is a mere reflection of parasite diversity
Source: Commun Biol. 2024 May 11;7:559. doi: 10.1038/s42003-024-06176-8 (PMC11088698; doi:10.1038/s42003-024-06176-8)
Supplement: Supplementary file 3 — Description of Additional Supplementary Files [file 42003_2024_6176_MOESM3_ESM.pdf]

Description of Additional Supplementary Files

File name: Supplementary Data

Description: Additional details for the results of the model selection, null model, LMM of host density and network analysis. In addition, it contains the numerical values for parasite species prevalence (Figure 2) and infection status prevalence (Figure 1).
