# Supplementary material for: Coinfection frequency in water flea populations is a mere reflection of parasite diversity
Source: Commun Biol. 2024 May 11;7:559. doi: 10.1038/s42003-024-06176-8 (PMC11088698; doi:10.1038/s42003-024-06176-8)
Supplement: Supplementary file 5 — Reporting Summary [file 42003_2024_6176_MOESM5_ESM.pdf]

nature portfolio

Corresponding author(s): Snir Halle

Last updated by author(s): Mar 17, 2024

Reporting Summary

Nature Portfolio wishes to improve the reproducibility of the work that we publish. This form provides structure for consistency and transparency in reporting. For further information on Nature Portfolio policies, see our Editorial Policies and the Editorial Policy Checklist.

Statistics

For all statistical analyses, confirm that the following items are present in the figure legend, table legend, main text, or Methods section.

n/a	Confirmed	
☐	☒	The exact sample size (*n*) for each experimental group/condition, given as a discrete number and unit of measurement
☐	☒	A statement on whether measurements were taken from distinct samples or whether the same sample was measured repeatedly
☐	☒	The statistical test(s) used AND whether they are one- or two-sided<br>*Only common tests should be described solely by name; describe more complex techniques in the Methods section.*
☒	☐	A description of all covariates tested
☐	☒	A description of any assumptions or corrections, such as tests of normality and adjustment for multiple comparisons
☐	☒	A full description of the statistical parameters including central tendency (e.g. means) or other basic estimates (e.g. regression coefficient) AND variation (e.g. standard deviation) or associated estimates of uncertainty (e.g. confidence intervals)
☐	☒	For null hypothesis testing, the test statistic (e.g. *F*, *t*, *r*) with confidence intervals, effect sizes, degrees of freedom and *P* value noted<br>*Give P values as exact values whenever suitable.*
☒	☐	For Bayesian analysis, information on the choice of priors and Markov chain Monte Carlo settings
☒	☐	For hierarchical and complex designs, identification of the appropriate level for tests and full reporting of outcomes
☐	☒	Estimates of effect sizes (e.g. Cohen's *d*, Pearson's *r*), indicating how they were calculated

Our web collection on statistics for biologists contains articles on many of the points above.

Software and code

Policy information about availability of computer code

Data collection	No software was used
Data analysis	Data analysis were done in Python 3.8 using the Pandas (McKinney 2010), NumPy (Harris et al. 2020), SciPy (Virtanen et al. 2020) and NetworkX (Hagberg et al. 2008) modules. These particularly included the null model construction, bootstrap, Pearson test and network analysis. GLMM, LMM and model selection were calculated in R 4.0.5 using lme4 (Bates et al. 2015) and MuMin (Barton 2022) packages, respectively, as well as the base packages. All the figures were constructed in Python 3.8 using the Matplotlib, Seaborn and NetworkX modules. The code is available on Figshare and can be accessed via https://doi.org/10.6084/m9.figshare.25407505.v1

For manuscripts utilizing custom algorithms or software that are central to the research but not yet described in published literature, software must be made available to editors and reviewers. We strongly encourage code deposition in a community repository (e.g. GitHub). See the Nature Portfolio guidelines for submitting code & software for further information.

Data

Policy information about availability of data

All manuscripts must include a data availability statement. This statement should provide the following information, where applicable:

- Accession codes, unique identifiers, or web links for publicly available datasets
- A description of any restrictions on data availability
- For clinical datasets or third party data, please ensure that the statement adheres to our policy

The datasets supporting is available on Figshare and can be accessed via https://doi.org/10.6084/m9.figshare.25407505.v1

Human research participants

Policy information about studies involving human research participants and Sex and Gender in Research.

Reporting on sex and gender	NA
Population characteristics	NA
Recruitment	NA
Ethics oversight	NA

Note that full information on the approval of the study protocol must also be provided in the manuscript.

Field-specific reporting

Please select the one below that is the best fit for your research. If you are not sure, read the appropriate sections before making your selection.

☐ Life sciences ☐ Behavioural & social sciences ☒ Ecological, evolutionary & environmental sciences

For a reference copy of the document with all sections, see nature.com/documents/nr-reporting-summary-flat.pdf

Ecological, evolutionary & environmental sciences study design

All studies must disclose on these points even when the disclosure is negative.

Study description	We studied the natural behavior of co-infection in natural population of Daphnia magna and their potential determinants. We sampled 8 ephemeral ponds inhabited by D. magna and dissected 100 individuals (when possible) from each sample to obtain presence\absence data if the different species of the parasite community within each individual host.
Research sample	8 ephemeral ponds along the Israeli coastal plain were sampled during one activity season (winter & spring). Each pond represent a single population of the host, Daphnia magna and their respective parasite communities.
Sampling strategy	On each sampling event, D. magna hosts were sampled by dragging a plankton net through the pond. The nets was dragged twice, once for 15 m (constant effort) to estimate the population density and a second time to retrieve a sample size of 100 adults. This sample size enable us to detect parasites when they are rare, therefor enable to monitor the seasonal changes more accurately. These samples were taken back to the lab for dissection and identification of parasites.
Data collection	From each sample that was brought to the lab, a random portion was placed on a petri dish and adult females of D. magna were identified and transfered to a jar. Then each of them was observed under a stereo microscope to identify disease phenotypes and the dissected and observed under a phase contrast light microscope for the identification of parasite spores. The presence\absence data of the different parasites within each host was recorded. All the handling of the animals in the lab as well as their identification and parasite screening was done by Snir Halle. The collection of the animals in the field was done jointly by Snir Halle, Ofir Hirshberg and Frida Ben-Ami
Timing and spatial scale	The samples were collected between February and June of 2019 and 2020 (4 ponds per year) as this is the activity season of Daphnia magna in Israel. The samplingin all the ponds began in February and ended when no D. magna could be found in two consecuitive samples. During February and March the ponds were sampled every other week while in April till the end of May they were sampled every week. Since the parasites in these ponds increase in abundance in the spring we chose to increase the samples frequency when the parasite community is expected to be more active. All the ponds were located within the same region of Israel, the coastal plain.
Data exclusions	We excluded all the samples that contained less than 20 adult hosts to avoid any bias that can be resulted from insufficient sample size. Due to this exclusion one of the ponds left with only two samples and was omitted from the analysis. The final data set contained 4,324 individuals from 7 populations.

Reproducibility	NA
Randomization	The animals were drawn randomly from the pond and later were taken randomly from the containers in which the samples were kept
Blinding	All the dissected animals were treated in the same manner.

Did the study involve field work? ☒ Yes ☐ No

Field work, collection and transport

Field conditions	The study was done during the Israeli winter and spring when the ponds are filled with water and D. magna is present till no D. magna could be found. The water temperature during our study ranged from 14.9 to 28.9
Location	Israels coastal plain. most southern coordinates (1°56'12.6"N 34°44'21.5"E) most northern coordinates (32°24'49.88"N 34° 53'53.06"E)
Access & import/export	The study sites were accessed by car, all the collected samples were transfered in plastic containers placed within a cooler to prevent them from over heating. No ethical approval was required to conduct this study. All samplings were conducted under permits 2019/42360 & 2020/42579 from the Israel Nature and National Parks Protection Authority.
Disturbance	All the sampling gear was washed in clean water and sterilized with ethanol to prevent the transmission of organisms between ponds during the sampling procedure.

Reporting for specific materials, systems and methods

We require information from authors about some types of materials, experimental systems and methods used in many studies. Here, indicate whether each material, system or method listed is relevant to your study. If you are not sure if a list item applies to your research, read the appropriate section before selecting a response.

Materials & experimental systems

n/a	Involved in the study	
☒	☐	Antibodies
☒	☐	Eukaryotic cell lines
☒	☐	Palaeontology and archaeology
☐	☒	Animals and other organisms
☒	☐	Clinical data
☒	☐	Dual use research of concern

Methods

n/a	Involved in the study	
☒	☐	ChIP-seq
☒	☐	Flow cytometry
☒	☐	MRI-based neuroimaging

Animals and other research organisms

Policy information about studies involving animals; ARRIVE guidelines recommended for reporting animal research, and Sex and Gender in Research

Laboratory animals	NA
Wild animals	Adults Daphnia magna were sampled from the ponds using a plankton net and were transfered within chilled plastic containers to the lab. In the lab the animals were dissected and screened for parasite spores (there is no nondestructive methods that enable to identify if hosts are infected by all the different members of the parasite community)
Reporting on sex	We dissected only adult females. Through most of the season D. magna reproduce via parthenogenesis and females comprises the vast majority of their population
Field-collected samples	The field samples were sorted in the lab and random portion of it was screened for D. magna. The randomly selected individuals were housed in 350 ml glass jars filled with artificial Daphnia medium. The rest of the sample housed in two litters glass jars filled with the original pond water and artificial Daphnia medium. The jars were placed in an experimental room with 12:12 L:D cycle and temperature of 20 degrees till further processing. all processing took place within 4 days from the sampling event.
Ethics oversight	No ethical approval was required to conduct this study. All samplings were conducted under permits 2019/42360 & 2020/42579 from the Israel Nature and National Parks Protection Authority.

Note that full information on the approval of the study protocol must also be provided in the manuscript.
